# Opportunities to investigate the effects of ivermectin mass drug administration on scabies

**DOI:** 10.1186/1756-3305-6-106

**Published:** 2013-04-17

**Authors:** Daniel Engelman, Diana L Martin, Roderick J Hay, Olivier Chosidow, James S McCarthy, L Claire Fuller, Andrew C Steer

**Affiliations:** 1 Centre for International Child Health, University of Melbourne, Royal Children’s Hospital Melbourne, Flemington Road, Parkville, Melbourne 3052, Australia; 2 Centers for Disease Control and Prevention, Atlanta, USA; 3 International Foundation for Dermatology, London, UK; 4 Department of Dermatology, AP-HP, Hôpital Henri Mondor, Université Paris-est Créteil Val de Marne, Créteil, France; 5 Queensland Institute of Medical Research, Brisbane, Australia; 6 Chelsea and Westminster Hospital, London, UK; 7 Murdoch Childrens Research Institute, Melbourne, Australia

**Keywords:** Scabies, Ivermectin, Mass drug administration, Neglected tropical disease

## Abstract

The recent article by Mohammed *et al.* demonstrates an impressive effect of ivermectin mass drug administration for lymphatic filariasis on the burden of scabies. Partnering scabies research within the evaluation and monitoring of Neglected Tropical Disease programmes could potentially increase our understanding of the epidemiology and control of scabies and its important bacterial complications.

## 

We read with interest the article by Mohammed *et al.* in the December 21st issue of *Parasites & Vectors*[1]*.*

The authors assessed the impact of an annual ivermectin mass drug administration (MDA) programme for lymphatic filariasis (LF) in Zanzibar, Tanzania, on the burden of scabies, using what they term a “rapid assessment” methodology. This involved retrospective review of health clinic records for the number of patients diagnosed and treated for scabies. The study observed a reduction of 68-98% in scabies cases over a five year period, indicating that annual MDA with ivermectin appears to have a significant effect on the burden of scabies in the community. Importantly, the reduction was also observed in children aged less than 5 years, who were not included in the ivermectin programme.

Whilst the study did not aim to show causation, the data represent some of the best available evidence demonstrating the effects of MDA programmes on other neglected tropical diseases (NTDs), and are the first indication of the potential impact on scabies. Understanding these additional effects is extremely important for the optimal design of integrated NTD control strategies, yet they have been overlooked in programmes to date.

Scabies is frequently complicated by bacterial skin infection (impetigo) which, in turn, can lead to a number of disease processes that carry significant morbidity, including severe soft tissue infections, invasive streptococcal and staphylococcal disease, acute post-streptococcal glomerulonephritis and possibly rheumatic heart disease [2]. Global efforts toward the control of scabies are particularly important to reduce the underappreciated morbidity and mortality of these secondary bacterial complications. Initial data suggest that MDA with ivermectin can not only reduce scabies prevalence, but reduce the burden of bacterial skin infection and indirect markers of renal damage (haematuria) [3].

There is a very large potential for increasing our understanding of the epidemiology and control of scabies through a research partnership, linking scabies with existing NTD control programmes. A number of simple measures introduced, as part of NTD monitoring and MDA evaluation, could provide invaluable information on the impact of ivermectin MDA on scabies and bacterial complications.

Whilst we applaud the authors for the publication of the rapid assessment method as an alternative to tests for parasite detection, a more direct evaluation of scabies prevalence would provide a better understanding of the effect of ivermectin MDA on disease burden. This could involve an annual cross-sectional prevalence study for scabies and impetigo in sentinel areas receiving ivermectin MDA. The International Alliance for the Control of Scabies (IACS) is working toward development and validation of protocols and case definitions for the diagnosis of scabies and impetigo, which are needed for both clinical and public health settings. Larger population samples would be required to examine the effect of ivermectin MDA on scabies complications such as invasive bacterial infection, renal and cardiac disease, but these are all feasible given the scope of current MDA programmes; for example the LF programme treated more than 500 million people in 2011 [4].

As now well established in the London Declaration [5], through creative collaborations outside of traditional disease-focussed programmes, comprehensive prevention and control strategies can yield increased benefits across a range of health outcomes for the most disadvantaged global populations.

## Abbreviations

LF: Lymphatic filariasis; NTD: Neglected tropical disease; MDA: Mass drug administration.

## Competing interests

The authors declare they have no competing interests.

## Authors’ contributions

All authors helped to draft the manuscript, and have read and approved the final manuscript.

## Authors’ information

All authors are members of the International Alliance for the Control of Scabies (IACS).
